# Evans syndrome associated with sterile inflammation of the central nervous system: a case report

**DOI:** 10.1186/1752-1947-7-262

**Published:** 2013-12-03

**Authors:** Ole Jan Simon, Tanja Kuhlmann, Stefan Bittner, Carsten Müller-Tidow, Jochen Weigt, Heinz Wiendl, Sven G Meuth

**Affiliations:** 1 Department of Neurology, University of Muenster, Muenster, Germany; 2 Institute of Neuropathology, University of Muenster, Muenster, Germany; 3 Department of Medicine A, Hematology, Oncology and Pneumology, University of Muenster, Muenster, Germany; 4 Department of Gastroenterology, Hepatology and Infectious Diseases, University of Magdeburg, Magdeburg, Germany

**Keywords:** Central nervous system, Evans syndrome, Hemolytic anemia, Immune thrombocytopenia, Inflammation

## Abstract

**Introduction:**

Evans syndrome is a rare hematological disease commonly defined as Coombs-positive hemolytic anemia and immune thrombocytopenia. Pathophysiology of this disease involves decreased cluster of differentiation (CD)4+ T-helper cell counts, increased CD8+ T-suppressor cell counts, a decreased CD4/CD8 ratio, and reduced serum immunoglobulin G, M and A levels - indicating a complex immune dysregulation. Association with other autoimmune diseases has been described although involvement of the central nervous system has not been reported so far.

**Case presentation:**

We here present a case of a 28-year-old woman of Turkish origin with progressive, disseminated, partly mass-forming lymphoplasmacellular infiltration (CD3+ and CD138+ cells) of the brain in association with Evans syndrome. No other central nervous system disorder could be identified on neuropathological evaluation. Although treatment with rituximab was effective to normalize erythrocyte and thrombocyte levels in her peripheral blood, it failed to dampen the inflammation in her central nervous system or prevent clinical progression. Initiation of treatment with cyclophosphamide resulted in stabilization of her central nervous system inflammation and the disease course.

**Conclusions:**

The complex immune dysregulation resulting in the antibody-mediated pathologies that can be regarded as the cause of both lymphoplasmacellular encephalitis and Evans syndrome renders this association to be of clinical relevance for both neurologists and hematologists. Our experience also sheds light on the effectiveness of different treatments for both disorders and we advise clinicians to take a closer look when encountering a combination of peripheral blood diseases with affection of the central nervous system.

## Introduction

Evans syndrome (ES) is a rare hematological disease commonly defined by the combination of simultaneous or sequential autoimmune hemolytic anemia (AIHA) and immune thrombocytopenia (ITP) sometimes associated with neutropenia in the absence of known underlying etiology. Robert Evans first described this syndrome in 1951, when he presented evidence for a spectrum-like relationship between acquired hemolytic anemia and primary thrombocytopenic purpura [1]. Nearly all presented studies so far have involved small numbers of patients, and interpretation of the results is made more difficult by the recent recognition that some cases of ES may instead have been autoimmune cytopenias secondary to autoimmune lymphoproliferative syndrome [2]. Whereas ES relies on antibody-mediated autoimmune hemolytic anemia and immune thrombocytopenia, the combination of AIHA and ITP can also be observed in patients with systemic lupus erythematosus, suggesting a link to other inflammatory mechanisms [3]. In addition, not all authors adhere to the strict definition of ES and include all patients with the combination of ITP and AIHA, irrespective of the etiology. However, summarizing all studies dealing with ES, there is convincing evidence to support dysregulated cellular and humoral immunity in the etiology of the disease.

Persistently decreased cluster of differentiation (CD)4/CD8 ratio in peripheral blood resulting from reduced CD4^+^ and increased CD8^+^ T cell levels has been described in six affected children compared to healthy controls and patients with chronic ITP [4]. These findings were corroborated in a 12-year-old patient with ES [5], in whom the reduced CD4/CD8 ratio persisted after splenectomy. In the same patient, an analysis of cytokine secretion before splenectomy revealed spontaneous T helper cell 1-type and T helper cell 2-type cytokine production and complete suppression of transforming growth factor β. After splenectomy, the patient had normalized levels of interleukin-2, interleukin-4, interleukin-10, transforming growth factor β and interferon γ, accompanied by clinical remission and increased numbers of natural killer cells. Of note, their patient received danazol (a derivative of the synthetic steroid ethisterone, a modified testosterone, also known as 17-alpha-ethinyl testosterone) before and after splenectomy. The effects of this substance on reported immune parameters cannot be excluded. Based on these findings, the authors hypothesized that spontaneous secretion of T helper cell 1-type cytokines (for example, IFN-γ) could explain the activation of autoreactive B-cells, which produce antibodies against red blood cells and platelets.

Although these results supported other studies demonstrating reduced serum immunoglobulin (Ig)G, IgM and IgA levels in patients with ES, pointing towards dysregulated cellular und humoral immunity [4], the significance of these abnormalities is uncertain as they also occur in other autoimmune conditions and in association with viral infection. Association with different autoimmune diseases, such as systemic lupus erythematosus [3], autoimmune hepatitis [6], Hashimoto’s thyroiditis [7], dermatomyositis [8] and chronic inflammatory demyelinating polyneuropathy [9], has also been described. We here present the case of a patient with ES in combination with dysregulation of autoimmune networks, resulting in central nervous system damage. To the best of our knowledge, such an association has not yet been reported.

## Case presentation

**Figure 1:**
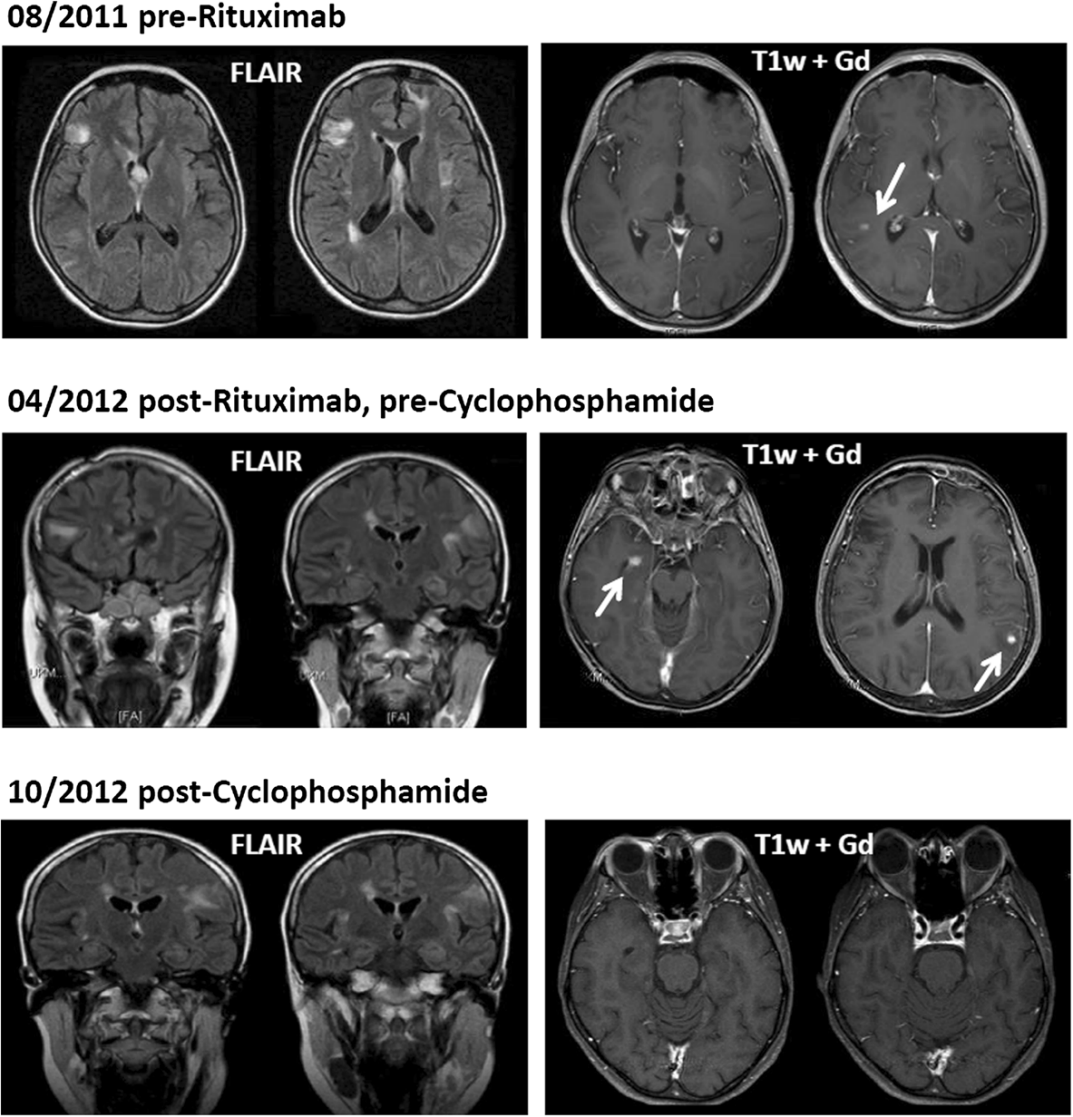
**Magnetic resonance imaging time course of the patient during different stages of treatment.** Fluid-attenuated inversion recovery and T1-weighted with gadolinium (exemplary images are shown). Arrows indicate gadolinium-enhancing lesions. Treatment with cyclophosphamide resulted in stable disease without new contrast enhancing lesions. FLAIR, fluid-attenuated inversion recovery; Gd, gadolinium.

In 1999, a 14-year old girl of Turkish origin presented with vertigo and generalized tonic-clonic seizures. Magnetic resonance imaging (MRI) analyses showed multiple hyperintense lesions in T2-weighted images (bi-hemispheric periventricular white matter, frontal cortex, left insular cortex, left brainstem). A symptomatic treatment with valproate was initiated. In 2002, three years after onset of central nervous system (CNS) inflammation, a mild thrombocytopenia (110 × 10^3^ cells/μL) occurred for the first time, but no further diagnostics were carried out. In the next years, she experienced various neurological symptoms such as hypoesthesia, weakness of her right arm, vertigo and double vision. These relapses occurred in a frequency of one to six events per year. MRI analyses showed a slow progression of inflammatory gadolinium-enhancing (Gd+) lesions, mostly localized in subcortical and periventricular areas (Figure 1). Conventional angiographies (2004 and 2008) revealed no signs of vessel stenosis, sinus thrombosis or other signs indicative of primary CNS vasculitis. Under the assumption of a relapsing-remitting multiple sclerosis, these clinical symptoms as well as future ‘relapses’ were treated with intravenous steroids until 2011.

**Figure 2:**
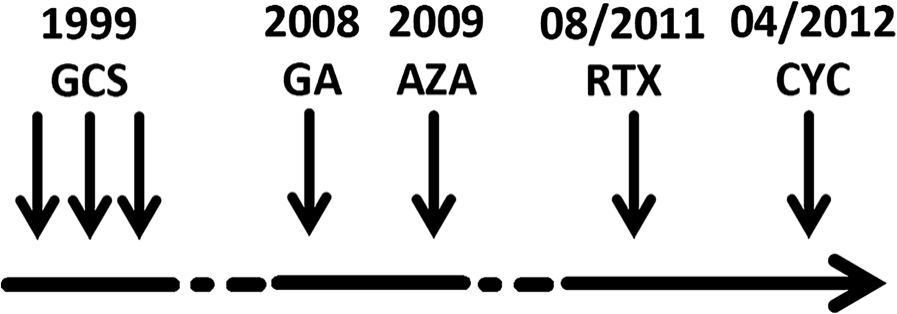
**Timeline of treatment.** AZA, azathioprine; GA, glatiramer acetate; GCS, glucocorticosteroids intravenous or orally; RTX, rituximab; CYC, cyclophosphamide.

Initiation of immune prophylactic immunomodulatory therapy was refused by our patient until 2008. In this year, immune modulatory treatment with glatiramer acetate was initiated, but was terminated by the patient after two weeks because of needle phobia. In 2009, the patient presented with additional anemia. After detection of warm agglutinins and a positive Coombs test, a diagnosis of ES was established. To exclude differential diagnoses, antinuclear antibodies, double-stranded DNA and rheumatoid factor were analyzed. Low serum levels of IgG and IgA allowed a retrospective diagnosis of common variable immunodeficiency, a condition often occurring in combination with ES [10]. Investigation of peripheral blood T cell subsets (CD4^-^/CD8^-^, CD3^+^, T-cell receptor αβ^+^) by flow cytometry was used to differentiate from autoimmune lymphoproliferative syndrome [2]. Treatment was initiated with oral administered corticosteroids and azathioprine. The latter needed to be stopped due to elevated liver enzymes (Figure 2).

After our patient’s first presentation to the clinic in 2011, an extensive clinical re-evaluation was performed due to increasing doubts concerning the initial diagnosis of multiple sclerosis. Analyses of cerebrospinal fluid (CSF) showed lymphocytic pleocytosis, intrathecal synthesis of IgG and IgM, impairment of blood-brain-barrier integrity and positive oligoclonal bands (June 2011: 52cells/μL, 44lymphocytes/μL, 1110mg/L protein, 10 oligoclonal bands (8 isolated in the CSF), 73.52% CD4^+^ T cells (normal 73.84 ±9.48%), 6.80% CD8^+^ T cells (normal 22.40 ±9.29%), CD4/CD8 ratio 10.9 (normal 3.81 ±1.52), 11.87% CD19^+^ B cells (normal 0.79 ±0.79%), 0.24% plasma cells (normal 0.042 ±0.042%)). A synopsis of results of clinical chemistry and CSF analysis during the time course is shown in Table 1.

**Table 1:** Laboratory results from peripheral blood and cerebrospinal fluid during treatment

**Peripheral blood**	**Normal range**	**Oct 2009**	**Jun 2011**	**Aug 2011**	**Nov 2011**	**Apr 2012**	**Sep 2012**	**Oct 2012**
**Thrombocytes** (10^3^/μL)	150 to 390	73.00	101.00	62.00	165.00	195.00	171.00	178.00
**Erythrocytes** (10^6^/μL)	3.92 to 5.08	1.43	4.36	4.44	4.74	4.88	4.38	4.18
**Leucocytes** (10^3^/μL)	4.49 to 2.68	7.51	4.71	5.74	5.00	6.42	4.60	4.35
**Lymphocytes** (10^3^/μL)	1.2 to 3.0		800.00		594.00	426.00	592.00	
**CD3+** (10^3^/μL)	0,8 to 1,2		1,049		0,475	0,300	0,601	
**CD4+** (%)	29 to 59		35.31		43.00	36.90	41.40	
**CD8+** (%)	19 to 48		34.13		31.00	31.30	54.25	
**CD4/CD8 ratio**	0.7 to 2.8		1.03		1.40	1.20	0.76	
**CD19+** (10^3^/μL)	0,1 to 0,8		0,144		0.00	0.00	0,04	0.00
**Hemoglobin**	12 to 16 [g/dL]	6.60	13.90	13.00	13.40	14.40	13.40	13.00
**IgG** (g/L)	7 to 16		3.20			4.41	0.98	
**IgA** (g/L)	0.7 to 3.8		0.10			0.05	0.04	
**IgM** (g/L)	0.4 to 2.8		0.90			0.20	0.14	
**Glucose** (mg/L)	70 to 120		92.70			88.10	97.30	
**Albumin** (g/L)	35 to 55		46.30			41.90	39.70	
**Oligoclonal bands**	<3		2.00			1.00	1.00	
**Cerebrospinal fluid**	**Normal range**	**Oct 2009**	**Jun 2011**	**Aug 2011**	**Nov 2011**	**Apr 2012**	**Sept 2012**	**Oct 2012**
**Lymphocytes** (/μL)	<4		44.00			13.00	1.00	
**CD3+** (%)	79.67 to 95.03		83.71			94.32	55.06	
**CD4+** (%)	64.36 to 83.32		73.52			87.97	65.52	
**CD8+** (%)	13.11 to 31.69		6.80			8.82	34.48	
**CD4+ CD8+** (%)	0.55 to 2.91		0.44			0.57	1.15	
**CD4/CD8 ratio**	2.29 to 5.33		10.79			9.94	1.67	
**CD4+ HLA DR +** (%)	2.71 to 9.71		10.69			14.67	5.55	
**CD8+ HLA DR +** (%)	15.74-25.60		16.12			50.59	44.83	
**CD19+ %** (%)	0 to 1.58		11.87			3.52	5.06	
**CD19+ CD138+** (%)	0 to 0.107		0.24			0.10	0.00	
**Lactate** (mmol/L)	>2.1		1.05			1.28	1.39	
**IgG** (mg/L)	<40		65.40			4.50	3.34	
**IgM** (mg/L)	<1		4.30			1.41	0.25	
**IgA** (mg/L)	<6		2.10			12.70	1.13	
**Glucose** (mg/dL)	60 to 85		35.80			53.60	49.20	
**Protein** (mg/L)	150 to 450		1.100.00			254.00	169.00	
**Albumin** (mg/L)	100 to 300		539.00			157.00	116.00	
**Oligoclonal bands**	<3		10.00			6.00	0.00	

*CD* cluster of differentiation, *Ig* immunoglobulin.

**Figure 3:**
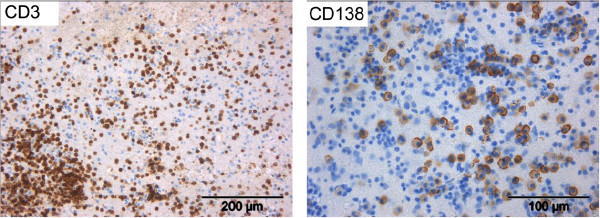
**Immunohistology of brain biopsy.** Example images show infiltration of T cells (CD3) and plasma cells (CD138), respectively. CD, cluster of differentiation.

In 2011, 16 lesions were evident on T2-weighted and fluid-attenuated inversion recovery images, with three new Gd + lesions localized in her right cerebellar hemisphere, right frontobasal region and right frontal cortex. Measurements of visual, sensory and motor evoked potentials showed no abnormalities. A differential diagnostic, including serological, microbiological and virological analyses from both her blood and CSF, revealed no signs for a systematic vasculitis, infection or any underlying specific autoimmune diseases. After another relapse presenting with unsystematic vertigo, we ultimately clarified the nature of her inflammatory leukoencephalopathy by performing a brain biopsy. This showed severe perivascular inflammatory infiltrates as well as diffuse cellular infiltration within the parenchyma. The infiltrates were dominated by small mature CD3^+^ T cells and high numbers of CD138^+^ plasma cells (Figure 3) whereas only low B-cell numbers could be observed. Plasma cells expressed lambda and kappa light chains. The proliferative index was between 5% and 7%. Immunohistochemical stainings for different pathogens (herpes simplex virus 1 and 2, Epstein-Barr virus, JC virus and toxoplasma) were negative. Additional stainings revealed no signs of demyelination, vasculitis or a brain-derived neoplasia.

**Figure 4:**
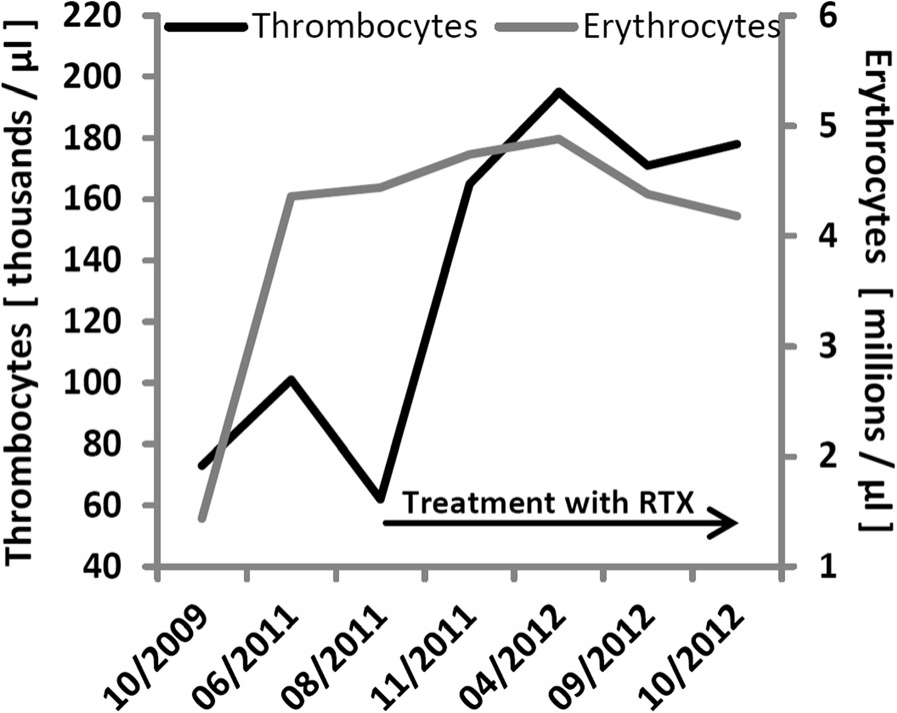
Parameters of cerebrospinal fluid and peripheral blood during treatment.

A diagnosis of lymphoplasmacellular encephalitis was made; inflammatory demyelinating disorders, vasculitis, neoplasias or infections were excluded. Based on the detection of CD20^+^ B cells and CD138^+^ plasma cells both by flow cytometry assessment of her CSF and by immunohistochemical stainings, we initiated treatment with anti-CD20 monoclonal antibody rituximab. This treatment regimen could be beneficially used in CNS inflammation and has already been reported to be effective in cases of ES [11,12]. Treatment was effective in terms of peripheral B cell depletion and led to the stabilization of her blood cell counts (Figure 4, Table 1). Despite the ongoing peripheral depletion of B cells, our patient developed new symptoms and two new Gd + MRI lesions eight months after starting treatment (April 2012; Figure 1). CSF analysis still showed elevated cell counts and normal protein levels (13cells/μL, 13 lymphocytes/μL, 254mg/L protein, intrathecal synthesis of IgG and IgM, five isolated oligoclonal bands, 87.97% CD4+ T cells, 0.57% CD8+ T cells, CD4/CD8 ratio 9.94, 3.5% B cells, 0.1% plasma cells; for details see Table 1). Although treatment with rituximab was effective in her ‘periphery’, it was not able to fully reduce B- and plasma cell counts in the CNS as reflected by CSF analysis (Figure 4). Therefore, we initiated treatment with cyclophosphamide at a dosage of 350mg/m^2^ on three consecutive days, followed by monthly intravenous applications of 600 to 750mg/m^2^, adapted to leukocyte levels. Cyclophosphamide is an alkylating immunosuppressant widely used for the treatment of cerebral vasculitis. Over 12 months of follow-up, this therapy has maintained a stable disease course and MRI findings.

## Conclusions

Patients with ES often benefit from treatment with corticosteroids, but they frequently experience hematological relapses. Second-line therapies consist of various immunosuppressants, among them methotrexate and mycophenolate mofetil. Splenectomy is another option in steroid-refractory or relapsing courses. There have been recent publications of cases treated with rituximab [6,11,13], which showed promising clinical effects. Rituximab is a monoclonal antibody directed against CD20 on B lymphocytes that leads to a reduction of B cells in the peripheral blood as well as putatively in the CSF [14]. The efficiency of an anti-CD20 therapeutic strategy further reinforces the concept of plasma cell-originated antibodies as a major pathophysiological mechanism of ES.

ES, thought of as a disease caused by immune dysregulation, has been associated with different autoimmune diseases such as systemic lupus erythematosus [3], dermatomyositis [8] and many others. To the best of our knowledge, an association with autoimmune mediated CNS inflammation has not been described so far. One case of an infant with ES associated with a polyclonal lymphoproliferative disease and subsequent parenchymal CNS lesions has been published [15]. Our case suggests a common underlying pathophysiology of both diseases (lymphoplasmacellular encephalitis and ES), mediated by antibodies derived from peripheral or CNS-infiltrating plasma cells, respectively. In the case we present here, progressive lymphoplasmacellular infiltrations of her CNS, resulting in multiple inflammatory lesions without demyelination or signs of a lymphoma or neoplasia, preceded the manifestation of ES. Our extensive differential diagnostic workup was not able to elucidate any underlying pathology for both diseases. The histological findings from a brain biopsy could not be ascribed to any other defined inflammatory CNS disease.

Although it is not possible to prove a direct link between lymphoplasmacellular encephalitis and ES, it seems likely since both diseases are based on complex immune dysregulation in the periphery and/or in the CNS. One can argue that peripheral dysregulation of immune responses, which leads to self-reactive antibodies directed against peripheral blood cells on the one hand and neuronal antigens on the other hand, can result in both peripheral and central manifestations and therefore represents a link for both diseases. It is therefore beneficial to conduct a complete neurological assessment including MRI and CSF examination in cases of patients with ES presenting with neurological symptoms, in particular cortical and cognitive manifestation. Dysfunction of the immune system can be observed in patients with ES in terms of peripheral CD4/CD8 alterations. In this case, we also observed an unusual disturbance of CD4/CD8 ratio in the CSF. Treatment with the B-cell-depleting antibody rituximab was able to stabilize the peripheral part of the disease while her intrathecal immune reaction was only partially reduced in terms of CD19^+^, CD4^+^, CD8^+^, CD4^+^ human leukocyte antigen DR^+^ and CD8^+^human leukocyte antigen^+^ cell counts. Treatment with cyclophosphamide further reduced these parameters, parallel to improved MRI findings and stabilization of the clinical disease course. Our findings support the notion that treatment with rituximab might in some cases be less effective for suppression of CNS inflammation compared to treatment strategies with cyclophosphamide.

## Consent

Written informed consent was obtained from the patient for publication of this case report and any accompanying images. A copy of the written consent is available for review by the Editor-in-Chief of this journal.

## Abbreviations

AIHA: Autoimmune hemolytic anemia; CD: Cluster of differentiation; CNS: Central nervous system; CSF: Cerebrospinal fluid; ES: Evans syndrome; Gd + : Gadolinium enhancing; Ig: Immunoglobulin; ITP: Immune thrombocytopenia; MRI: Magnetic resonance imaging.

## Competing interests

The authors declare that they have no competing interests.

## Authors’ contributions

OJS treated the patient, analyzed the data and wrote the manuscript. TK performed the histopathological analysis of the CNS material. SB treated the patient and analyzed the data. CMT, JW and HW treated the patient. SGM treated the patient and wrote the manuscript. All authors read and approved the final manuscript.
